# Hypomorphic Mutations in the BCR Signalosome Lead to Selective Immunoglobulin M Deficiency and Impaired B-cell Homeostasis

**DOI:** 10.3389/fimmu.2018.02984

**Published:** 2018-12-18

**Authors:** Christoph B. Geier, Kai M. T. Sauerwein, Alexander Leiss-Piller, Isabella Zmek, Michael B. Fischer, Martha M. Eibl, Hermann M. Wolf

**Affiliations:** ^1^Immunology Outpatient Clinic, Vienna, Austria; ^2^Clinic for Blood Group Serology and Transfusion Medicine, Medical University of Vienna, Vienna, Austria; ^3^Department for Health Science and Biomedicine, Danube University Krems, Krems, Austria; ^4^Biomedizinische Forschungs GmbH, Vienna, Austria; ^5^Medical School, Sigmund Freud Private University, Vienna, Austria

**Keywords:** primary immunodeficiency, B-cell defects, selective IgM deficiency, BTK, BLNK, marginal-zone B cells, natural antibodies

## Abstract

B cell activation via the B cell receptor (BCR) signalosome involves participation of signaling molecules such as BTK and BLNK. Genetic defects in these molecules are known to impair B cell differentiation and subsequently lead to agammaglobulinemia. Here we identified novel mutations in BTK and BLNK in two unrelated patients that perturb the intrinsic B-cell receptor signaling pathway and lead to selective IgM deficiency, whereas production of other immunoglobulin isotypes and IgG antibody response remain intact. Currently it is unknown how BCR signaling strength affects mature B cell development in humans. Both patients show reduced levels of BCR signalosome phosphorylation as well as impaired BCR-dependent Ca^2+^ influx, which was accompanied by a marked decrease in IgD^+^IgM^+^CD27^+^ MZ-like B-cells. We further describe reduced expression of essential B cell differentiation factors such as BAFF-R and T-Bet in the patients' B-cells, which might contribute to the observed deficiency of MZ-like B cells. MZ-like B cells are known to produce natural IgM antibodies that play an essential role in immune homeostasis. By using surface plasmon resonance (SPR) technology and a synthetic blood group A trisaccharide as antigen we were able to show that both patients lack the presence of anti-blood group A IgM considered to be prototypical natural antibodies whereas IgG levels were normal. Antibody binding dynamics and binding affinity of anti-blood group A IgG were comparable between patients and healthy controls. These results indicate that human IgM deficiency can be associated with signaling defects in the BCR signalosome, defective production of natural IgM antibodies in the blood group A/B/0 system and abnormalities in B cell development.

## Introduction

The B cell receptor (BCR) induces B-cell activation and differentiation following antigen exposure. Membrane bound immunoglobulins and the non-covalently bound CD79a/b (Iga/b) form the BCR complex (1). After ligand dependent BCR aggregation tyrosine residues in the cytoplasmic ITAM portion of CD79a and CD79b are phosphorylated by spleen tyrosine kinase (Syk). This phosphorylation recruits the BCR signalosome to amplify the activation, including the kinases Syk, Lyn, and Bruton tyrosine kinase (Btk), the guanine exchange factor Vav, and the adaptor proteins Grb2 and B-cell linker (BLNK) (2).

B lymphocyte homeostasis depends on tonic and induced BCR signaling. BCR signaling strength is a major driver of the developmental fate to facilitate the production and maintenance of immunocompetent pools of mature follicular (Fo) BI and FoBII cells and marginal zone (MZ) B cells while remaining self-tolerant (3). A recent study by Tsiantoulas et al. identified secreted IgM as a major regulator for BCR-signaling strength and proper B cell development in mice by acting as a negative regulator of BCR signaling (4). Immunoglobulin M plays a crucial role in the adaptive immune system, as it appears early in the course of an infection and bridges the gap between innate immunity and production of high affinity IgG (5). Beside its well-documented protective role against invasive pathogens, natural IgM plays a crucial role in immune homeostasis and immune development (5–8). A significant proportion of serum IgM consists of naturally occurring IgM, which is produced independently of exposure to foreign antigens and without the need for T helper cells. While it is clear from studies in mice that natural IgM is produced by B1 cells at birth, in humans the cellular origin of natural IgM still remains controversial. An orthologous human B-1 cell population has not been clearly identified, and the existence and phenotype of a human B cell subset responsible for production of natural IgM still remains debated (9–13).

Isolated deficiency of IgM was first described in 1967 by Hobbs and colleagues in two children with low to absent levels of serum IgM and fatal meningococcal meningitis (14). Selective Immunoglobulin M deficiency (sIgMD) is characterized by isolated low to absent levels of serum IgM and defective IgM antibody response following vaccination, infection or natural exposure, while the number of peripheral blood B lymphocytes, immunoglobulin isotype class switch, and serum levels of other immunoglobulins and IgG antibody responses are intact in the majority of patients (15–17). The clinical manifestation of sIgMD comprises a heterogenous spectrum ranging from bacterial infections in the majority of patients to atopic or autoimmune disease manifestation in otherwise asymptomatic patients (14–16). Complete selective IgM deficiency is considered a rare primary immunodeficiency with a reported prevalence of 0.03% in a community-based study (18). However, the prevalence of patients with decreased to borderline-detectable IgM is estimated to be higher, up to 2.1% in selected cohorts (19–21).

In this study we sought to identify the molecular pathomechanism leading to selective IgM deficiency. We wanted to further clarify the role partial BCR signaling defects might have in B cell development and homeostasis. Murine models on how BCR signaling strength influences MZ and FO B cell development are contradictory and data in humans are scarce (22, 23). We identified novel hypomorphic BTK and BLNK mutations that dampen BCR signaling strength in two unrelated male patients with sIgMD. We demonstrated that in these patients reduced BCR signaling is associated with impaired formation of natural IgM antibodies of the blood group AB0 system and abnormalities in MZ B-cell development, possibly due to altered expression of essential MZ-B cell differentiation factors BAFF-R and T-bet.

## Materials and Methods

### Determination of Serum Immunoglobulins and Antibodies

Serum concentrations of immunoglobulins and IgG subclasses were determined by standard laser nephelometry on a Siemens nephelometric analyzer (Siemens Healthcare; Germany) using reagents purchased from Siemens-Behring Division. IgG and IgM antibodies against bacterial and viral antigens were determined using commercially available enzyme-linked immunosorbent assay (ELISA) kits [IgG antibodies against tetanus and diphtheria toxoid, tick borne encephalitis (TBE) virus, Haemophilus influenza type b (Hib)] or an in-house produced isotype-specific ELISA (IgG and IgM antibodies against 23-valent pneumococcal capsular polysaccharide) as previously described (24).

### DNA Isolation and Targeted Resequencing

Genomic DNA was prepared from peripheral blood by spin column purification (QIAamp DNA Blood Mini Kit; QIAGEN, Germany). Targeted resequencing of 222 primary immunodeficiency genes listed in the 2011 IUIS expert committee report and candidate genes was performed for the two index patients. (Table S1) Nextera Custom Enrichment kit was used according to standard protocols (Illumina, USA). Targeted DNA library was quantified and validated using Illumina Eco Realtime (Illumina; USA) and Agilent Bioanalyzer (Agilent Technologies; USA). The library was sequenced in a multiplex pool on a single (151 bp paired-end reads) Miseq flowcell (Illumina, USA). Data analysis was performed using CLC Genomic Workbench (QIAGEN, Germany).

### cDNA Preparation and Gene Expression of IgM Splice Forms

Total RNA was isolated using RNeasy Mini Kit (Qiagen, Netherlands) and Oligo dT primed cDNA library was prepared using SuperScript IV First-Strand Synthesis System (Thermo Fisher Scientific, USA) from an EBV-transformed lymphoblastoid cell line (EBV-LCL) from patients and healthy controls. Alternative spliced transcript PCR was performed to visualize different forms of IgM (precursor, secreted, and membrane) using Phire Hot Start II DNA Polymerase (Thermo Fisher Scientific; USA). Amplicons were visualized using standard LE-Agarose electrophoresis (Biozym, Germany).

### Amplification-Refractory Mutation System (ARMS)

The coding sequence of BLNK (cDNA) and BTK (gDNA) was amplified using Phire Hot Start II DNA Polymerase (Thermo Fisher Scientific; USA). Allele-specific PCR was used to characterize BLNK Pro110Ala and Ala158Ser alleles. Each allele (wild type and mutant form) was amplified separately with an allele-specific primer in combination with a general primer using Maxima Hot Start Taq DNA Polymerase (Thermo Fisher Scientific, USA). All the resulting amplicons were purified and custom Sanger sequenced (Eurofins Genomic; Germany). Results were aligned to BLNK NM_013314.3 and BTK NG_009616.1 sequences as reference using CLC GenomicWorkbench (QIAGEN, Germany).

### Western Blot and SDS Page

Epstein-Barr-virus transformed lymphoblastoid cell line of patient A, patient B and a healthy control were lysed for 30 min in ice-cold RIPA lysis buffer system (Santa Cruz Biotechnology, USA), and insoluble material was removed by centrifugation (16,000 × g, 10 min, 4°C). Twenty Microgram of protein were resolved in either 8%SDS-polyacrylamide gel electrophoresis (SDS-PAGE) or in 4% Native-polyacrylamide gel electrophoresis. Samples were subsequently electro transferred onto a polyvinylidene difluoride membrane (Immobilon-P; Millipore), and immunoblotted with anti-IgM antibody (2C12-3) (Santa Cruz Biotechnology Inc; USA). Detection was performed using the SuperSignal West Pico ECL detection system (Thermo Scientific; USA).

### Biacore® Surface Plasmone Resonance (SPR)

A Biacore® T200 device (kindly provided by Florian Koelle, GE Health Care) was used to determine the concentration and affinity of blood-group A antibodies. For this a contact time of 600 s and a flow rate of 10 μl/min was chosen. Flow cell one (FC-1) was immobilized without trisaccharides and served as blank during binding analysis. FC-2 was immobilized with 1 mg/ml blood group A or B trisaccharide amine derivative (Dextra Laboratories Ltd., Reading, UK).

For binding analysis, serum was diluted by half with HBS-EP and injected in FC-2 for 180 s at a flow rate of 10 μl/min. The flow-path was 2–1. To determine IgM and IgG levels of blood group A- or B-bound antibodies, anti-human IgG Abs [polyclonal aHIgG (γ-chain), Sigma-Aldrich, USA] and anti-human IgM [polyclonal aHIgM (μ-chain), Sigma-Aldrich, USA] were injected for 180 s at a flow rate of 10 μl/min directly after the serum sample. The difference in resonance units (ΔRU) between report point “stability” and “baseline” was indicative for the amount of bound antibody, ΔRU between “enhance_baseline” and “enhance_level” represents the isotype-specific portion of bound antibodies. The chip was regenerated twice with 50 mmol NaOH for 30 s at a flow rate of 10 μl/min with a stabilization period of 5 s after the second regeneration to reach the same baseline as prior to the measurement.

For kinetic measurement, an association time of 200 s and dissociation time of 800 s was chosen. To analyze the curve exponential decay was assumed and the half-life (t1/2) and the dissociation constant kd of the antigen-antibody complex were calculated according to equation 1 and 2.

t(1/2)=((t1)/(ln2(N(t)/(N(0)))

Equation 1 Half-life of antigen-antibody complex

$$\begin{array}{l} \left(\text{N}0-\text{N}\left(\text{t}\right)\right)/\text{Δ}t^{*}N0=-kd \end{array}$$

Equation 2 Dissociation constant kd of antigen-antibody complex

t0: time (s) at start of decay, t1: time (s) at end of decay, N0: RU at start of decay, N(t): RU at end of decay, t1/2: half-life (s), Δt: time of dissociation, kd: dissociation constant

The amount of anti-A or anti-B antibody that associated with the corresponding blood group A or B trisaccharide immobilized on the sensor chip surface was obtained by subtracting the FC-II value (RU) from the FC-I value (RU).

### Flow Cytometry

Flow cytometry was performed as previously described (25). Peripheral venous blood was collected in EDTA containing tubes from patients with selective IgM deficiency and 14 healthy blood donors that served as controls. B cell subsets were characterized as follows; Naïve (CD19^+^IgD^+^CD27^−^), Transitional(CD19^+^CD27^−^CD24^high^CD38^high^), Follicular (CD19^+^CD27^−^CD24^dim^CD38^dim^), MZ (CD19^+^CD27^+^IgD^+^IgM^+^), Class Switched (CD19^+^CD27^+^IgD^−^IgM^−^), IgM-only (CD19^+^IgD^−^IgM^+^), CD21low (CD19^+^IgM^+^CD21^low^CD38^low^), and Plasmablasts (CD19^+^CD27^++^CD38^++^). All values are expressed as percent of total peripheral CD19^+^ B cells. Supporting Information Table S2 shows the monoclonal fluorophore-conjugated antibodies used. Dead cells were excluded and at least 100,000 events within the “lymphogate” were acquired. Cells were acquired with a FACSVerse (Becton Dickinson; USA) using standard protocols and analyzed using FACSuite software (Becton Dickinson; USA).

### Analysis of B-cell Function

Human peripheral blood mononuclear cells (PBMCs) and LCL-EBV cell lines were isolated and generated as previously described (25). PBMC or LCL- EBV cells were transferred in 24-well flat-bottomed plates and were cultured in complete RPMI medium supplemented with 10% fetal bovine serum. 1 × 10^6^ cells per well were stimulated for 4 days at 37°C and 5% CO2 using 20 μg/mL goat ${\text{F}}_{\left(\text{a}{\text{b}}^{\text{′}}\right)}$2 anti–human IgM and IgD, each (Sigma-Aldrich, USA). Intracellular staining and Calcium influx was performed as previously described (26, 27). B cells were identified by CD19^+^ and upregulation of activation marker was calculated by subtracting the geometric mean fluorescence intensity of unstimulated B-cells from the geometric mean of fluorescence intensity of activated B-cells. Supporting Information Table S2 shows the monoclonal fluorophore-conjugated antibodies used.

### Statistical Analysis

Statistical comparisons between experiments performed in cells from healthy controls and repeat experiments with cells from the two patients were performed by calculating the Mann Whitney *U*-test using Prism Graphpad 4.0 software. Statistically significant differences obtained in intergroup comparisons were confirmed by Kruskal–Wallis one-way analysis of variance using Prism Graphpad 4.0 software. Values of *p* < 0.05 were considered as significant, (ns statistically not significant, ^*^p ≤ 0.05, ^**^p ≤ 0.01.

## Ethics Statement

The study was conducted in accordance with the Declaration of Helsinki and fulfills the guidelines of the Austrian Agency of Research Integrity (OeAWI). Patients gave their informed consent that anonymized data collected as part of the routine medical attendance (immunological analysis, flow cytometry analysis, and genetic mutation analysis) could be included in a scientific publication. All patient information in this study is anonymized and de-identified prior to analysis, and only anonymized and de-identified patient information is contained in this study. Samples used for genetic and molecular non-clinical analyses in this study were derived from leftover material obtained as part of the routine medical attendance the patients received. No extra intervention was carried out. With respect to the genetic and molecular non-clinical analyses this study was approved by the Ethics Committee of the Immunology Outpatient Clinic as a study using the residual specimens biobank of the Immunology Outpatient Clinic. According to the Ethics Committee of the City of Vienna and the legal regulations to be applied (§15a Abs. 3a Wiener Krankenanstaltengesetz) no additional ethics committee evaluation is required for a non-interventional study using data collected as part of the routine medical care the patients received.

## Patient Characteristics

Patient A was a 15-year old male referred for immunological investigation because of IgM deficiency, subtle hypogammaglobulinemia, recurrent stomatitis aphthosa and recurrent respiratory tract infections such as sinusitis and bronchitis (Table 1). He suffered from pneumonia at the age of 6, but otherwise had an uneventful medical history. He was the child of healthy unrelated parents of Austrian origin, a healthy brother was 10 years old. Upon initiation of antibiotic prophylaxis with amoxicillin (50% therapeutic dose daily) and pneumococcal vaccination susceptibility to respiratory infections normalized.

**Table 1:** Immunological Phenotype of two patients with sIgMD.

	**Patient A**	**Patient B**	**Normal range**
**SERUM IMMUNOGLOBULIN LEVELS [mg/dl]**			
IgG	745	903	790–1,700
IgA	92	791	76–450
IgM	39	27	90–350
**SERUM IGG SUBCLASS LEVELS [mg/dl]**			
IgG1	501	460	500–880
IgG2	158	302	150–600
IgG3	43	75	20–100
IgG4	<6	50	8–120
**LYMPHOCYTE SUBPOPULATIONS (PERCENTAGE OF LYMPHOCYTES AND ABSOLUTE NUMBERS IN PARENTHESIS)**			
CD3^+^	68 (919)	80 (2,784)	53–85 (694–2,976)
CD4^+^	40 (541)	48 (1,670)	31–66 (386–2,022)
CD8^+^	28 (379)	28 (974)	21–43 (297–1,011)
CD19^+^	13 (176)	14 (487)	7–23 (71–549)
CD56^+^	17 (230)	10 (348)	6–29 (98–680)

Patient B was a 37-year old male of Turkish descent referred for immunological investigation by the treating nephrologists because of IgM deficiency. Asymptomatic renal insufficiency was detected at the age of 28 years when a cirrhosis of the left kidney and mild hydronephrosis of the right kidney were found. Serum creatinine was 3.2 mg/dl (normal range 0.6–1.2 mg/dl), proteinuria was 2.5 g/d. He reported no increased susceptibility to infections, and his chronic renal insufficiency caused only mild clinical symptoms (development of fatigue and tachycardia upon physical strain).

## Results

### Novel Hypomorphic BTK and BLNK Mutations in Two Unrelated Patients With Selective IgM-Deficiency

**Figure 1:**
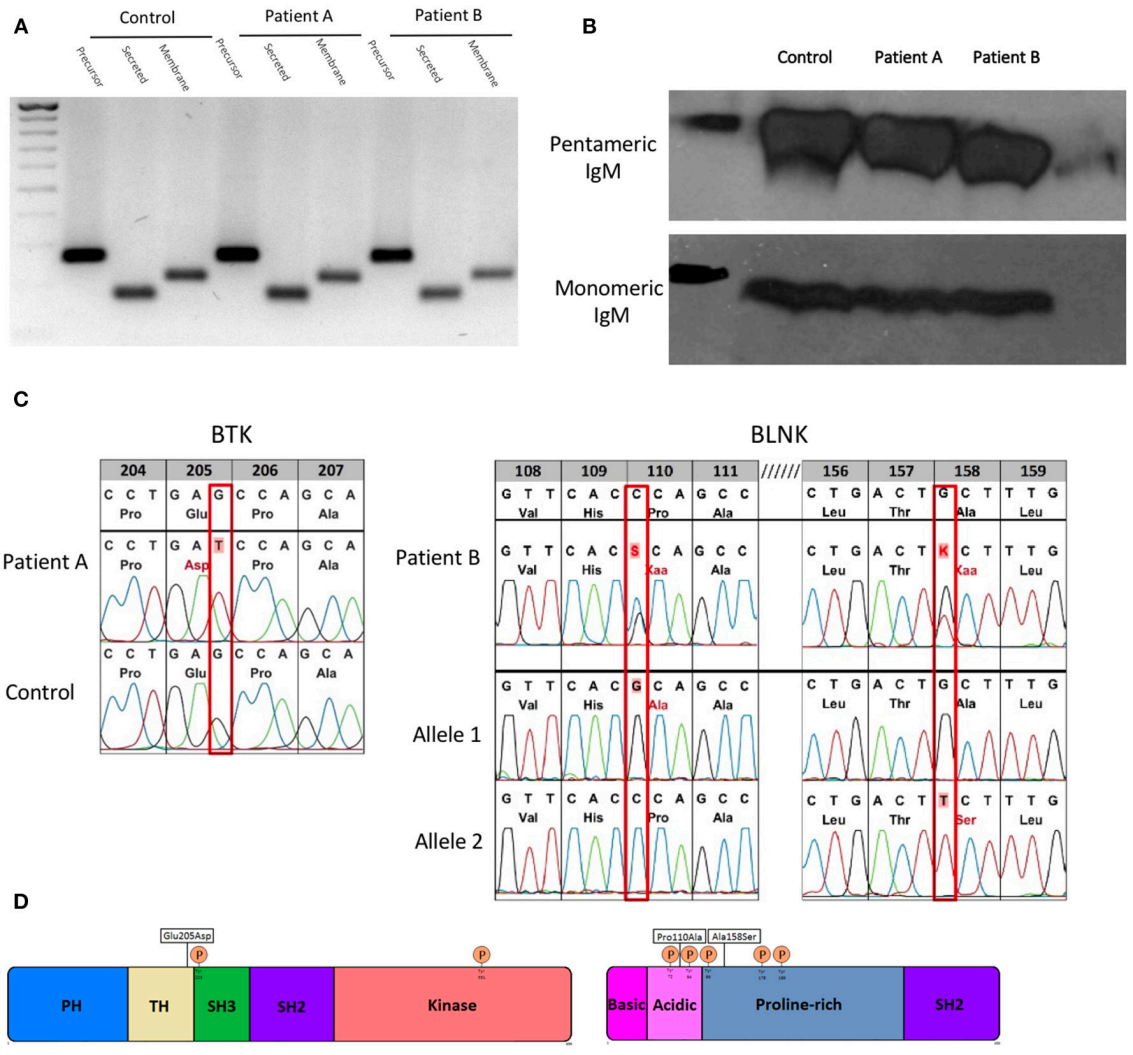
Molecular characterization of novel hypomorphic BTK/BLNK mutations. **(A)** Gene expression of precursor, secreted and membrane IgM of patients and control EBV-LCL quantified by semi-quantitative cDNA-PCR. **(B)** Protein expression of monomeric and pentameric IgM of patients and control EBV-LCLs by SDS-PAGE or native-PAGE and detected by western blot. **(C)** BTK/BLNK Mutation analysis of genomic DNA from peripheral blood. Patient A is hemizygote for a c615G>T in BTK. Patient B is compound heterozygous in BLNK for c328C>G, c472G>T. Healthy controls served as wild type control. **(D)** Schematic depiction of mutation sites and phosphorylation sites in BTK and BLNK.

The mRNAs encoding the membrane-bound and secreted immunoglobulin heavy chains are produced from identical primary transcripts, which are differently processed at their 3′ ends. Regulation of membrane-bound vs. secreted forms of the immunoglobulin heavy chains depends on the competition of 2 mutual cleavage polyadenylation sites (pAs/pAm) (28). In mice targeted deletion of the mu heavy chain cleavage polyadenylation site pAs leads to deficiency of secreted IgM with intact expression of surface IgM and normal secretion of other immunoglobulin isotypes (29). Therefore, we sequenced mu heavy chain gene including the polyadenylation sites in both patients with sIgMD and found no alterations (data not shown). Both patients' B cells were able to express precursor, secreted and membrane IgM mRNA (Figure 1A). Furthermore protein expression of monomeric and native pentameric IgM (Figure 1B) and surface expression of IgM on the B cell membrane (data not shown) was comparable to healthy controls.

To elucidate the genetic basis of the patients' selective IgM deficiency we used a targeted resequencing approach to sequence potential candidate genes. In both patients, we identified defects within the intrinsic B-cell receptor signaling pathway. Patient A harbored a c615G > T missense mutation in exon 8 in the tyrosine kinase BTK. The G > T transition resulted in a glutamic acid to aspartic acid substitution at position 205 within the highly conserved proline-rich (PRR) region located at the C-terminus of the TEC homology (TH) domain (Figures 1C,D). Proline rich regions are involved in protein-protein interactions, including interactions with G proteins and intramolecular association with the SH3 domain (2). Mutations within the proline rich regions have been shown to abolish SH3 domain binding and result in functional impairment of BTK, pointing toward a potential biologic relevance of the BTK mutation found in patient A (30).

Patient B harbored a biallelic mutation in BLNK, which was subsequent identified to be compound heterozygous by amplification-refractory mutation system (ARMS). Both mutations (c328C > G, pPro110Ala/c472G > T, pAla158Ser) are located within a functionally relevant region of the N terminus domain of BLNK (Figures 1C,D), in close proximity to highly conserved tyrosine residues which serve as a scaffold to assemble downstream targets like VAV, NCK, BTK, and PLCγ2 (31). Restriction fragment length polymorphism (RFLP) analysis of DNA of 200 unrelated individuals did not reveal BTK or BLNK mutations identical to that seen in our sIgMD patients (data not shown).

### Novel Hypomorphic BLNK and BTK Mutations Result in Impaired B-cell Receptor Signaling

Mutations within the BCR signalosome such as BTK or BLNK usually result in absent protein expression, a severe block of BCR signaling and an arrest at the pre-B cell stage, subsequently leading to agammaglobulinemia (32). Our patients had normal numbers of peripheral blood B cells and no agammaglobulinemia (Table 1).

**Figure 2:**
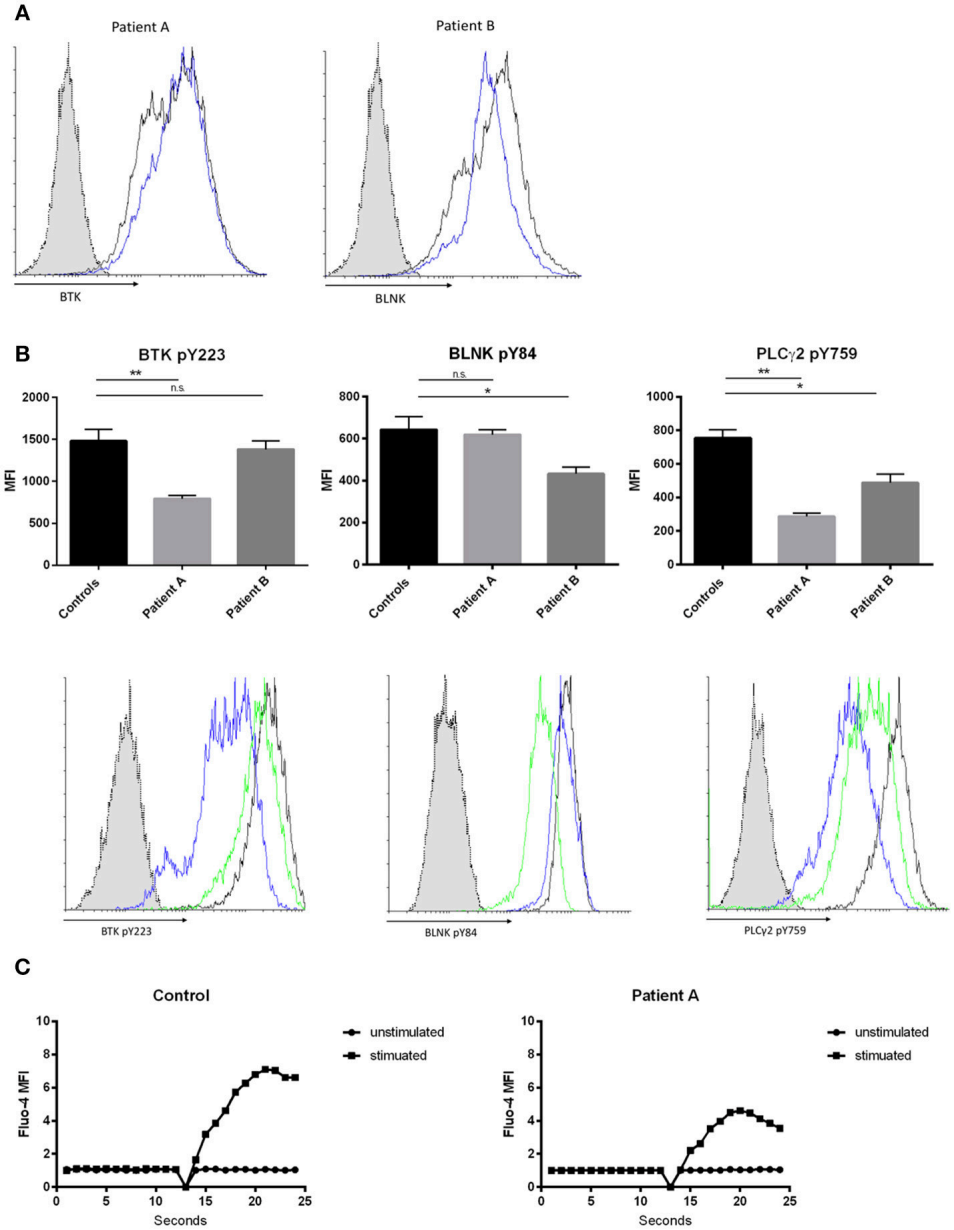
Effects of hypomorphic BTK/BLNK mutations on BCR signaling. **(A)** Representative FACS histograms depicting BTK (left) and BLNK (right) expression of αIgM/αIgD -stimulated EBV-LCLs. Blue histograms represent patients, black histograms represent healthy control and dotted gray histograms represent isotype control. **(B)** Bar graphs and representative flow cytometry plots showing the expression of pBtk, pBLNK, and pPLCγ2 in αIgM/αIgD -stimulated EBV-LCLs. Blue histograms represent patient A, green histograms represent patient B, black histograms represent healthy control, and dotted gray histograms represent isotype control. Results in bar graphs are expressed as mean fluorescence intensity (MFI, mean ± SD) of stimulated CD20^+^ EBV-LCLs after subtraction of expression of unstimulated CD20^+^ EBV-LCLs (no significant difference was found in basal expression between controls and patients, data not shown). CD20^+^ EBV-LCLs were stimulated with αIgM and αIgD antibodies. Bars represent the mean and standard deviation of five healthy controls and three repeat experiments using cells from the two patients. (*ns* = statistically not significant, ^*^p ≤ 0.05, ^**^p ≤ 0.01, Mann Whitney *U*-test) **(C)** Kinetics plot showing calcium influx of Fluo-4 loaded peripheral CD19^+^ B cells from patient A and healthy control following activation with αIgM and αIgD antibodies.

BTK and BLNK protein expression was not altered in both patients compared to healthy controls, when quantified by flow cytometry (Figure 2A). To assess whether novel BTK and BLNK mutations described are associated with B-cell signaling impairment we stimulated the patients' EBV transformed B-cells with αIgM and αIgD antibodies and analyzed phosphorylation of BTK and BLNK by flow-cytometry. Autophosphorylation of BTK at position Y223, located within the Src-homology 3 domain, was diminished in patient A as compared to healthy controls, suggesting a reduced SH3-mediated downstream signaling (Figure 2B, left panel).

Patient B did present with a reduction in BLNK phosphorylation at position Y84 compared to healthy controls examined in parallel. BLNK is a substrate for SYK, which phosphorylates Y84, and phosphorylated BLNK provides docking sites for various molecules including activated BTK and PLC gamma 2 (Figure 2B, middle panel). Activated BTK brought in proximity of PLC gamma 2 by BLNK leads to phosphorylation and activation of PLC gamma 2. Thus, we hypothesized that the hypomorphic mutations observed in BTK as well as in BLNK might impact PLC gamma 2 activation (33, 34). Phosphorylation of PLC gamma 2 at position Y759 was diminished in both patients' B cells stimulated via the BCR as compared to healthy controls examined in parallel (Figure 2B, right panel).

Intact BTK and BLNK activity is essential for normal BCR-dependent Ca^2+^ signaling in human B cells (33, 35). Therefore, we investigated whether the hypomorphic BTK mutation found in patient 1 results in impaired calcium flux through store operated calcium channels. Primary B-cell were loaded with Fluo-4 and activated with αIgM and αIgD. Patient A demonstrated a decreased influx of intracellular calcium, indicating abnormal function of store-operated calcium entry in this patient with selective IgM deficiency (Figure 2C).

### Impairment of BCR Signaling Is Associated With Skewed B-cell Homeostasis in Patients With Selective IgM Deficiency

**Figure 3:**
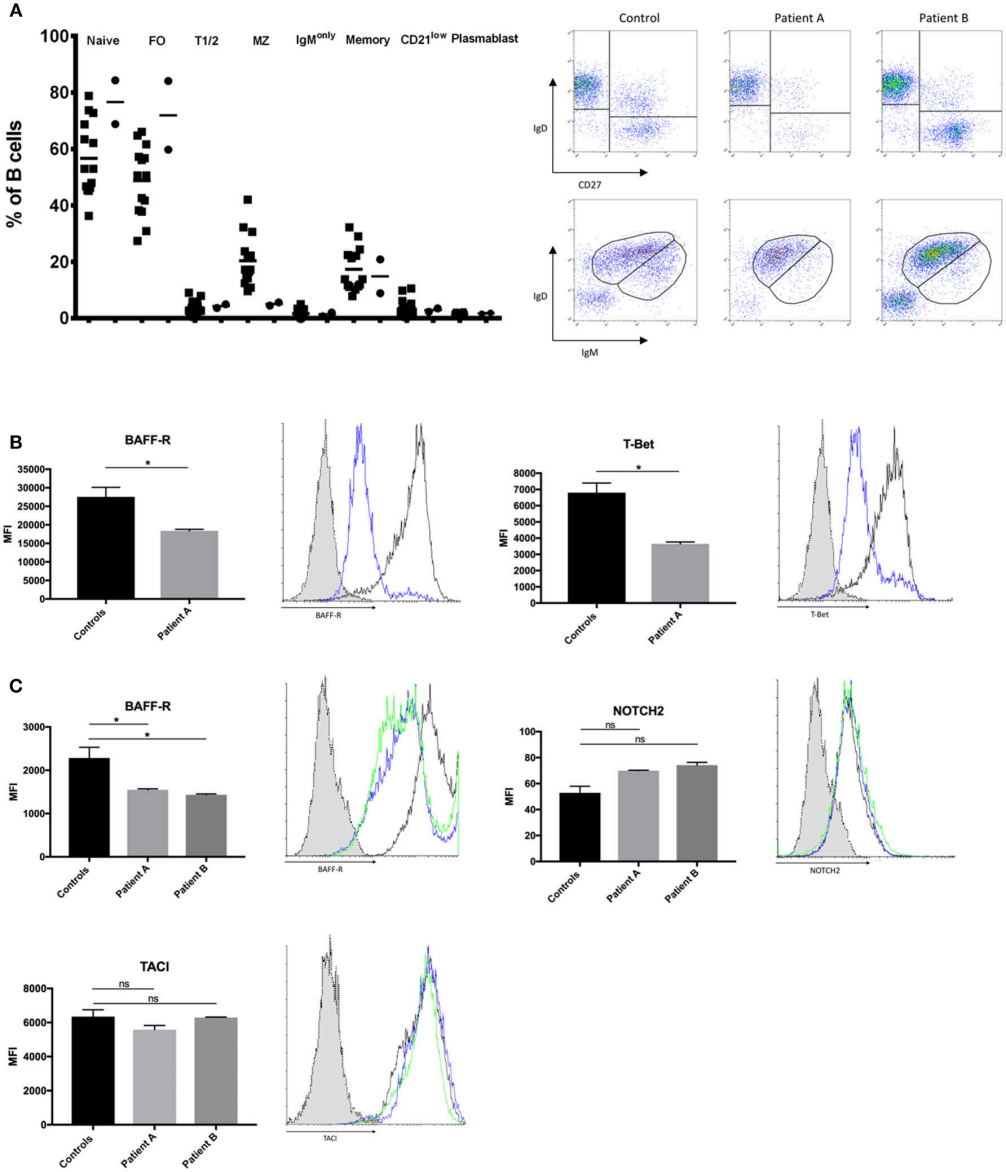
Altered B-cell compartment in sIgMD with impaired BCR signaling. **(A)** Bar graphs and representative flow cytometry plots showing percent of total CD19^+^B cells of Naïve (CD19^+^IgD^+^CD27^−^) Transitional T1/2 (CD19^+^CD27^−^CD24^high^CD38^high^) Follicular FO (CD19^+^CD27^−^CD24^dim^CD38^dim^) MZ (CD19^+^CD27^+^IgD^+^IgM^+^), Class Switched Memory (CD19^+^CD27^+^IgD^−^IgM^−^) IgM only (CD19^+^IgD^−^IgM^+^), CD21low (CD19^+^IgM^+^CD21^low^CD38^low^), and. Patients are depicted as filled circles (•) and healthy controls (*n* = 14) as filled squares (■), horizontal bars represent the mean. **(B,C)** Bar graphs and representative flow cytometry plots showing the expression of BAFF-R, T-Bet, NOTCH2, and TACI. Blue histograms represent patient A, green histograms represent patient B, black histograms represent healthy control and dotted gray histograms represent isotype control. Results are expressed as mean fluorescence intensity (MFI, mean ± SD) on stimulated peripheral CD19^+^ B-cells or stimulated CD20^+^ EBV-LCLs after subtracting expression of unstimulated CD19^+^ B-cells or stimulated CD20^+^ EBV-LCLs (no significant difference was found in basal expression between controls and patients, data not shown). Peripheral CD19^+^ B-cells **(B)** and CD20^+^ EBV-LCLs **(C)** were stimulated with αIgM and αIgD antibodies. Bars represent the mean and standard deviation of three experiments. *ns*, statistically not significant; ^*^p ≤ 0.05, Mann Whitney *U*-test.

BCR signaling strength is the major determinant of the developmental fate of mature B cells. However, the precise effect of BCR-signaling on the shaping of B cell homeostasis is not yet fully clarified. Recent studies in mice report that strong BCR signaling favors MZ B cell development while other studies report increased FO B cells (4). We were interested how impaired BCR signaling might affect B cell development in our patients with selective IgM deficiency. We found a significant decrease in the numbers of MZ B cells while follicular and naïve B cells were present in normal to increased levels (Figure 3A). Peripheral blood numbers of transitional stage B cells, class switched memory B cells, IgM only memory B cells and plasmablasts did not differ between healthy controls and patients. We further quantified levels of CD21low B cells, as previous reports in patients with common variable immunodeficiency (CVID) associated increased CD21low B cells with defects in calcium- dependent BCR-activation (36). However, we did not find alterations in the levels of CD21low B cells in our sIgMD patients (Figure 3A).

Recent evidence suggests that BCR/BTK signaling positively autoregulates crosstalk with BAFF-R, which is a fundamental developmental factor for survival and differentiation of MZ B cells (3). We therefore quantified levels of BAFF-R expression following BCR activation in peripheral B-cells of patient A and EBV-LCLs of both patients. BAFF-R expression in peripheral B-cells of patient A and EBV-LCLs of both patients were significantly reduced following BCR activation (Figures 3B,C). In addition to BCR/BAFF-R interaction, Notch signaling pathway is essential in the generation of MZ B cells. As Notch expression is independent of BCR signaling strength, we hypnotized that NOTCH2 expression is unaffected on patients' B cells. We found a slight increase in NOTCH2 expression on both patients' EBV-LCL after BCR activation, which might resemble an insufficient compensatory mechanism (Figure 3C). Low TACI expression has been described as a reason for the impaired T cell independent antigen response in XID mice (37). We therefore quantified the expression of TACI in BCR-activated EBV-transformed LCL, however there was no significant difference in TACI expression between patients and healthy controls (Figure 3C). We further quantified levels of T-bet in peripheral B-cells of patient A, as MZ B cells migrate in a T-bet dependent manner (38). We could observe a marked reduction in T-bet expression of patient A's peripheral B-cells (Figure 3B).

### Intact IgG Antibody Response in Patients With Selective IgM Deficiency

Furthermore, we were interested whether impaired BCR signaling and disturbed B-cell homeostasis alters T-cell dependent and T-cell independent antibody responses in patients with selective IgM deficiency.

**Figure 4:**
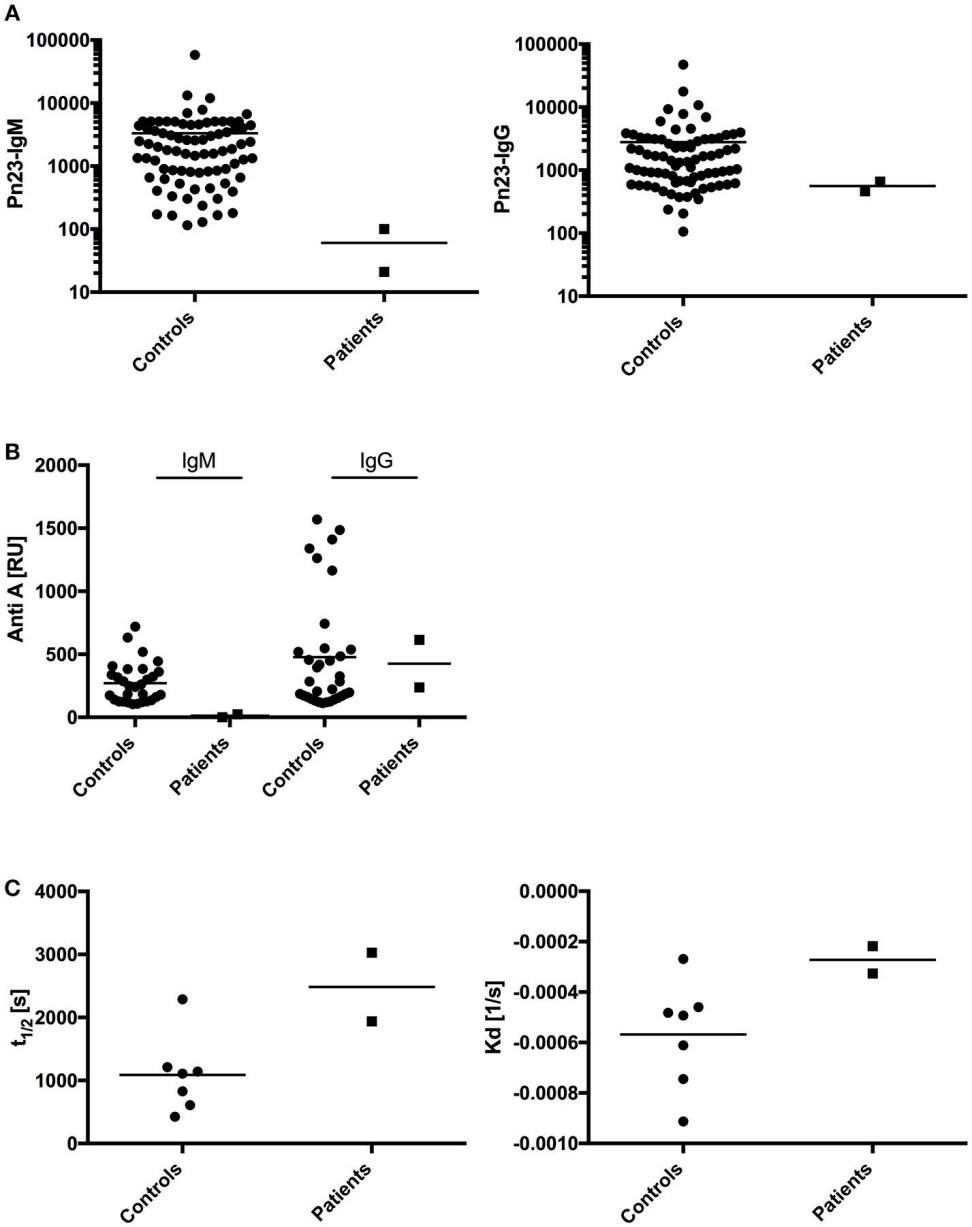
Analysis of antibody response in patients with impaired BCR signaling.**(A)** In healthy individuals (*n* = 80, circles) and patients A and B (*n* = 2, squares) antibodies against pneumococcal polysaccharides (anti-PnPs) were determined by ELISA and presented as IgM–Pn23-antibody response (left panel) or IgG–Pn23-antibody response (right panel). Healthy controls and patient A were immunized with the PnPs vaccine Pneumo 23 Vaccine “Pasteur Merieux” (Pn23), and blood samples were drawn 4–6 weeks after vaccination. **(B**) Serum samples were diluted in HES-EP buffer (1:2) and samples were injected over the blood group A trisaccharide-coupled CM5 sensor chip. Amounts of anti-A antibodies were recorded as sensorgrams in resonance units (RU) against time in FC-I and FC-II. **(C)** Estimated affinity constant of blood group specific anti-A IgG antibodies is calculated as KD and t_1/2_. Horizontal bars represent the mean.

T- dependent IgG antibody responses to protein antigens such as tick-borne encephalitis virus (TBEV), VZV, HAV, and tetanus toxoid were normal in both patients, as were IgG antibody titers against the capsular polysaccharide of Hib (Table 2; patient A received four childhood vaccinations with conjugated Hib vaccine, patient B's Hib-IgG were produced after natural exposure/infection). Both patients responded with normal levels of T-independent IgG titer when challenged with 23-valent unconjugated pneumococcal vaccine (patient A) or after natural exposure/infection (patient B) (Figure 4A, right panel). In contrast, both patients displayed a defective IgM responsiveness to T-independent bacterial polysaccharide antigens such as 23-valent pneumococcal capsular polysaccharides, either after vaccination with Pneumo 23 “Merieux” (patient A) or following natural exposure/infection (patient B) (Figure 4A, left panel), or tetravalent unconjugated meningococcal vaccine (Mencevax, patient A, data not shown).

**Table 2:** IgG antibody response to protein and polysaccharide antigens.

**Vaccine antigen**	**Antibody isotype**	**Patient A**	**Patient B**	**Normal range**
Tetanus toxoid	IgG (ELISA, IU/ml)	2.60	n.a.	>0.4
HAV	IgG (ELISA, IU/L)	3,971	6,743	>100
TBEV	IgG (ELISA, U/ml)	2,024	n.a.	>310
VZV	IgG (ELISA, VE)	22.2	29.7	>11
Hib	IgG (ELISA, ug/ml)	6.08	2.69	>1

In addition, we investigated how impaired BCR signaling in selective IgM deficient patients perturbs formation of natural antibodies. Blood group A/B antibodies are directed against carbohydrate epitopes that form the AB0 antigens on red blood cells (RBCs) and are considered prototypic natural antibodies (39, 40). We applied surface plasmon resonance (SPR) technology and synthetic blood group A trisaccharide as the antigen to investigate titers of IgM and IgG anti-A antibodies and real-time analysis of molecular binding dynamics. IgM anti-A antibodies were undetectable in both sIgMD patients. In contrast, IgG Anti-A antibodies were detectable in normal titers in patients compared to healthy controls (Figure 4B). Results were comparable when IgM and IgG titers of anti-blood group B antibodies were analyzed (data not shown).

To measure whether binding characteristics of anti-blood group A-IgG antibodies are altered in our patients, we analyzed the binding dynamics of IgG anti-A antibodies. We found no significant change in binding stability (halftime of antibody-antigen complex, Figure 4C left panel) and estimated affinity constant (K_d_, Figure 4C right panel).

## Discussion

The identification of BTK as the cause of X-linked agammaglobulinaemia (XLA) in 1993 provided the first description of a monogenetic gene defect causative of inherited B cell deficient agammaglobulinemia (41). Since then a pleiotropic spectrum of mutations within the BCR and BCR signalosome have been described. The majority of mutations described lead to a severe block in B-cell development, while reports of milder phenotypes are scarce (42). Hypomorphic mutations usually result in low numbers of circulating B cells, low residual levels of immunoglobulins and variable defects in IgG antibody formation (43–46). Lim LM and colleagues reported two siblings with selective IgM deficiency and a missense mutation in BTK leading to a severe reduction in circulating B cells similar to previous published hypomorphic BTK mutations (42, 47). Our findings confirm and extend previous publications by reporting novel mutations in BTK and BLNK in two unrelated sIgMD patients, associated with moderately impaired BTK and BLNK function and impaired BCR signaling, indicating a functional relevance compatible with a hypomorphic nature of these mutations. The novel BTK E206D mutation in patient A is located within the TH-domain. The TH-domain consists of two distinct motifs, an N-terminal Btk motif adjacent to the PH domain and a highly conserved proline rich region (PRR) located at the C-terminus (48). The N-terminal Btk motif is involved in Zn^2+^ binding and mutations in these residues result in altered protein folding and stability and thereby cause XLA (49, 50). The PRR motif occurs twice in BTK at residues 186–192 and 200–206. The PRR-TH regions of BTK mediates specific interactions with SH3-containing proteins and thereby is essential for intermolecular or intramolecular interactions and critical for biological signal transduction (30). Mutations that lead to instability and loss of BTK protein resulted in severe XLA whereas detection of reduced levels of protein is associated with decreased clinical severity (51). BTK levels in patient A harboring the E206D mutation were not altered when quantified by flow cytometry. We hypothesize that rather than destabilizing the BTK protein, E206D impairs BCR signalosome function.

To date, 6 patients with mutations in the scaffold protein BLNK have been described (52–55). These patients lack expression of BLNK as they harbor either nonsense or frameshift mutations, generally having clinical findings that are comparable to those seen in patients with mutations in BTK. We herein report the first case of biallelic missense mutations in BLNK, Pro110Ala and Ala158Ser, with normal expression of BLNK analyzed by flow cytometry. Upon phosphorylation, non-ITAM tyrosine residues of BLNK located within a proline-rich domain serve as scaffold by assembling the BCR signalosome (56, 57). Both of our novel biallelic mutations, Pro110Ala and Ala158Ser, are located in close proximity to highly conserved tyrosine residues. We could demonstrate that the biallelic mutations in patient B result in impaired BLNK function and therefore fail to amplify PLC γ -mediated signaling. Rather than abolishing B cell signaling and causing agammaglobulinemia, our data indicate that the hypomorphic mutations in patients with selective IgM deficiency described might hamper BCR signaling.

The majority of selective IgM deficiency cases occur sporadically and only a minority of patients are described to have an aberrant B cell phenotype similar to our patients described, thus selective IgM deficiency is likely a heterogeneous disorder (17, 58). In addition to hypomorphic mutations in BCR signalosome genes, other disease mechanisms could cause selective IgM deficiency. B cells from mice with a targeted deletion of the μs cleavage polyadenylation site (pAs) do not secrete IgM but are still capable of expressing surface IgM and IgD and secreting other Ig isotypes (59). Furthermore, aberrations in phosphorylation of the RNA polymerase II (RNAP-II) and recruitment and polyadenylation of CstF factors (CstF77, CstF64, CstF50) shifts the balance to the membrane form rather to the secreted form of IgM (28, 60).

Up to now it is unknown how BCR signaling strength influences MZ and FO B cell development and homeostasis in humans. Murine models are contradictory, as mice that lack BTK show reduced total numbers of circulating B cells, while MZ B cells seemed to be less affected than the FO population (61). On the other hand, it has been shown that the presence of self-antigen-specific BCR, thus leading to strong BCR signaling, favors MZ B cell development (22). We herein describe novel hypomorphic BTK and BLNK mutations that were associated with reduced BCR signaling and a pronounced reduction in MZ B cells and an expansion of FO B cells. Our findings are supported by Tsiantoulas and colleagues, who show that low dose Ibrutinib treatment, a kinase inhibitor targeting BTK, lowers BCR signaling and promotes FO and restricts MZ B cell formation (4).

Furthermore, we could show that in patients with hypomorphic BTK and BLNK mutations BAFF-R and T-Bet, essential MZ B cell homeostasis factors are reduced. Previous reports identified that BCR signalosome signaling constitutes a positive autoregulatory loop that mediates crosstalk between BCR and BAFF-R (3).

We cannot however totally exclude that the observed reduction in MZ B cell numbers in selective IgM deficiency is a secondary phenomenon due to the lack of circulating IgM. This explanation seems unlikely as it has been demonstrated that BCR signaling is increased in sIgM^−/−^ mice, and secreted IgM is known to negatively regulate BCR activation by acting as a decoy receptor for antigens that otherwise would be recognized by membrane-expressed BCR. Thus, the increased BCR signaling in sIgM^−/−^ mice favors MZ B cell and restricts FO B cell development (4). In addition, EBV transformation is known to alter B- cell function by expressing LMP2A a viral protein that mimics the B-cell receptor (62). Due to limited patient's material we had to conduct experiments in EBV-LCLs, further studies in primary B-cells need to be conducted to confirm the observed alterations in BCR signalosome function in primary cells.

There is no orthologous human B-1 cell population and the phenotype of a human B subset that secrets natural IgM still remains debated (9–13). We cannot definitely pinpoint the lack of natural IgM antibodies to the impaired numbers and homeostasis of MZ-B cells found in our patients. Follow up studies need to address whether possible newly defined B1- B cell subsets are impaired in patients with hypomorphic BCR signalosome mutations. In addition, as IgG response in our patients seems to be intact, future studies should address whether there is a difference in recruitment of BTK and BLNK between the cytoplasmic tails of IgM and IgG, e.g., with ImageStream analysis or by immunostaining, as the cytoplasmic tails of IgM BCR is shorter compared to IgG BCR (63).

In conclusion, our data indicate that selective IgM deficiency can be present in patients with hypomorphic BTK and BLNK mutations that dampen BCR signaling strength. We demonstrated that reduced BCR signaling is associated with perturbed MZ B-cell development and might impair formation of natural IgM antibodies in the blood group A/B/0 system by altering expression of essential MZ-B cell differentiation factors such as BAFF-R and T-bet.

## Author Contributions

CG designed the research, performed experiments, interpreted and analyzed the results, and wrote the manuscript; KS, AL-P, and IZ performed experiments, interpreted, and analyzed the results; MF and ME: interpreted and analyzed results; HW took overall responsibility for the research performed in this study and guided the writing of the manuscript. All authors have read and approved the contents of the manuscript and are accountable for all aspects of the work.

### Conflict of Interest Statement

The authors declare that the research was conducted in the absence of any commercial or financial relationships that could be construed as a potential conflict of interest.

## Supplementary Material

Table S1 Primary immunodeficiency genes sequenced.

Table S2 Monoclonal fluorophore conjugated antibodies for flow cytometry.
