# Supplementary material for: Hypomorphic Mutations in the BCR Signalosome Lead to Selective Immunoglobulin M Deficiency and Impaired B-cell Homeostasis
Source: Front Immunol. 2018 Dec 18;9:2984. doi: 10.3389/fimmu.2018.02984 (PMC6305442; doi:10.3389/fimmu.2018.02984)
Supplement: Table S1 — Primary immunodeficiency genes sequenced. [file Table_1.docx]

| ACTB | CD79A | IKZF1 | NEMO | STAT3 |
| --- | --- | --- | --- | --- |
| ADA | CD79B | IL10 | NFKBIA | STAT5A |
| ADAM17 | CD81 | IL10RA | NHEJ1 | STAT5B |
| AICDA | CD8A | IL10RB | NHP2 | STIM-1 |
| AIRE | CEBPE | IL12B | NKX2-5 | STK4 |
| AK2 | CFD | IL12RB1 | NLRP12 | STX11 |
| AP3B1 | CFH | IL-17F | NLRP3 | STXBP2 |
| APOL-I | CFI | IL-17RA | NOD2 | TAP1 |
| ATM | CFP | IL1RN | NOP10 | TAP2 |
| BLM | CIITA | IL2RA | NRAS | TAPBP |
| BLNK | COLEC10 | IL2RG | ORAI1 | TAZ |
| BTK | COLEC11 | IL36RN | PIGA | TBK1 |
| C16orf57 | COLEC12 | IL7R | PIK3R1 | TBX1 |
| C1QA | CORONIN1A | IRAK2 | PLCG2 | TERC |
| C1QB | CSF2RA | IRAK4 | PLDN | TERT |
| C1QC | CTSC | IRF8 | PMS2 | TINF2 |
| C1R | CXCR4 | ISG15 | PNP | TLR3 |
| C1S | CYBA | ITCH | PRF1 | TMC6 |
| C2 | CYBB | ITGB2 | PRKCD | TMC8 |
| C3 | DCLRE1C | ITK | PRKDC | TNFRSF13B |
| C4A | DCLRE1C | JAK3 | PSTPIP1 | TNFRSF13C |
| C4B | DiGeorge | LCK | PTPRC | TNFRSF1A |
| C5 | DKC1 | LIG1 | RAB27A | TRAC |
| C6 | DNMT3B | LIG4 | RAC2 | TRAF3 |
| C7 | DOCK8 | LPIN2 | RAG1 | TRIF |
| C8A | ELANE | LRBA | RAG2 | TYK2 |
| C8B | FADD | LRRC8A | RFX5 | UNC119 |
| C9 | FAS | LYST | RFXANK | UNC13D |
| CARD11 | FASLG | MAGT1 | RFXAP | UNC93B1 |
| CARD9 | FCN3 | MASP1 | RHOH | UNG |
| CASP10 | FERMT3 | MASP2 | RMRP | VPS13B |
| CASP8 | FOXN1 | MBL2 | RNF168 | WAS |
| CD19 | FOXP3 | MCM4 | ROBLD3 | WIPF1 |
| CD21 | FPR1 | MEFV | RPSA | XIAP |
| CD247 | G6PC3 | MRE11 | SBDS | ZAP70 |
| CD27 | GATA2 | MS4A1 | SERPING1 | ZBTB24 |
| CD3D | GFI1 | MSH5 | SH2D1A |  |
| CD3E | GPD | MSN | SLC35C1 |  |
| CD3G | HAX1 | MVK | SLC37A4 |  |
| CD40 | ICOS | MyD88 | SMARCAL1 |  |
| CD40L | IFNGR1 | NBN | SP110 |  |
| CD46 | IFNGR2 | NCF1 | SPINK5 |  |
| CD59 | IGHM | NCF2 | STAT1 |  |
| CD70 | IGLL1 | NCF4 |  |  |
| CD74 | IKBKB |  |  |  |
|  | IKBKG |  |  |  |
